# CHALLENGES AND OPPORTUNITIES OF (RE)CONNECTING AFTER COVID-19

**DOI:** 10.1093/geroni/igad104.0237

**Published:** 2023-12-21

**Authors:** Amy Rauer, Katherine Fiori

## Abstract

Although social distancing and other COVID-19 precautions were designed to protect older adults, the resultant disruptions to social connections were considerable. As vaccines became widely available, however, older adults were able to reconnect and resume social activities, a process we refer to in this symposium as social reintegration. The following studies highlight the challenges and opportunities associated with this reintegration process. First, Fuller et al.’s longitudinal, mixed-methods study considers how older adults’ coping strategies changed over the course of the pandemic as social distancing precautions were lifted. Building off this paper, Rauer et al. explore how and with whom older adults stayed occupied while reintegrating, and how activity profiles changed across six months. Using the same dataset to investigate the nature of these social interactions and the role of personality, Fiori et al. examine how positive and negative social exchanges and optimism predict changes in older adults’ attitudes about how COVID-19 shaped their family life. The role of individual differences is underscored by So et al.’s study of the surprisingly dynamic links between older adults’ generativity and their mental health as they reintegrated over six months. The transitory nature of this reintegration period is further highlighted by Marini et al., who found that older adults anticipated a wide variety of stressors and rewards over this time that changed with the emergence of new COVID-19 variants. Together, these papers paint a compelling picture of how older adults navigate times of transition to reconnect to others and establish a “new normal”.

